# Exploring the Biocontrol Potential of Fungus *Alternaria gaisen* GD-011 in the Tibetan Plateau

**DOI:** 10.3390/plants14030331

**Published:** 2025-01-23

**Authors:** Haixia Zhu, Huan Li, Yongqiang Ma

**Affiliations:** 1 Academy of Agriculture and Forestry Sciences, Qinghai University, Xining 810016, China; 2 State Key Laboratory of Plateau Ecology and Agriculture, Qinghai University, Xining 810016, China; 3 Key Laboratory of Agricultural Integrated Pest Management of Qinghai Province, Xining 810016, China

**Keywords:** *Alternaria*, crop safety, herbicidal activity, infection process

## Abstract

In screening for biocontrol strains with broad-spectrum and efficient herbicidal activity, strain GD-011 isolated from naturally susceptible *M. sativa* (*Medicago sativa* L.) roots was selected as a promising candidate. The control effects of strain GD-011 on nine weeds of Qinghai farmland were evaluated in vitro and in vivo, and its safety to five local crops was tested. The in vivo spray inoculation test showed that strain GD-011 had a strong pathogenic effect on the weeds *M. verticillata* (*Malva verticillata* L.), *E. densa* (*Elsholtzia densa* Benth), and *A. fatua (Avena fatua* L.), with incidence rates of more than 80.87% and fresh weight control effects of more than 71.8%. Crop safety tests showed that the *B. napus* (*Brassica napus* L.) crop is moderately susceptible to strain GD-011, with slight reactions in *H. vulgare* (*Hordeum vulgare* L.), but strain GD-011 is safe for *T. aestivum* (*Triticum aestivum* L.), *P. sativum* (*Pisum sativum* L.), and *V. faba* (*Vicia faba* L.) Observations on the morphological characteristics combined with a sequence analysis of the ribose rDNA internal transcribed spacer (rDNA ITS), the Elongation factor (*EF-1α*) gene, and the antigen-related protein gene (*Alt a1*) identified strain GD-011 as *Alternaria gaisen*. Scanning electron microscopy observations showed that the mycelia of strain GD-011 invaded the leaf tissue through the stomata on the surface, with the formation of a parasitic mycelium network on the surface of the tissue, the metabolism of *E. densa* leaf tissues was disturbed, and leaf tissues appeared to be broken. From the perspective of its herbicidal potential, the metabolites of strain GD-011 have good control effects on most weeds and are relatively safe for crops.

## 1. Introduction

Weeds cause massive losses in agricultural production, and their natural characteristics include strong adaptability and resistance [1]. The proliferation of weeds limits the utilization of water and nutrients by crops. Weed infestation is the main challenge in maintaining a high crop yield, as weeds compete with crops for nutrients, water, and sunlight, and occupy the growth space of food crops, leading to poor food crop development and greatly reducing crop yields [2,3]. The traditional methods of weed control include manual, mechanical, and chemical weeding techniques [4]. With the development of social civilization and the improvement of public health awareness, the development of new broad-spectrum, efficient, and low-toxicity microbial herbicides and biological weeding technologies have become major trends.

Microbial herbicides refer to microbial preparations that are developed and prepared from fungi, bacteria, and viruses, with the most widely studied being from natural plant pathogenic fungi [5]. After contacting the weeds, fungi will penetrate the plant surface and enter the plant tissues to form infective structures, which will spread in the plants in large numbers, affecting the physiological processes of the weeds [6]. After some live strains invade plants, their metabolites can poison the plants, weaken plant defense systems, or cause plant infection and death [7,8]. Another type of pathogenesis mainly involves toxins or antibiotic substances produced by microorganisms, including peptides, macrocyclic herbicides, and other herbicides. When these herbicides enter the plant body, they can damage its internal structures, including important processes such as photosynthesis, as well as protein and lipid metabolism [9]. So far, the research on microbial herbicides involving fungi is far more extensive than that of other species. More than 40 genera of fungi have the potential to be developed as biological herbicides, mainly *Alternaria*, *Fusarium*, *Botrytis*, *Hymenospora*, *Cavitospora*, *Sclerospora*, *Cercospora*, *Anthracnose*, *Sclerotinia*, *Phoma*, *Puccinia*, and *Scleromonospora.* About 80 active microbial species, such as those in the genus *Sphaelomyces*, have shown the ability to control over 70 types of weeds [10,11,12].

*Alternaria* spp. fungi have been widely used as an important biological resource for the control of pests, diseases, and weeds in the field [10]. Reports indicate that *Alternaria* spp. can produce numerous secondary metabolites, such as ATX-I, ATX-II, TeA, AAL, ALT, ACT, and others, and these toxins have shown strong inhibition of weed growth and germination [13,14,15]. For example, the toxin TeA extracted from *Alternaria* inhibits photosynthesis in *Eupatorium adenophorum*, thereby hindering the transport of photosynthetic electrons. Toxin AAL isolated from *Alternaria* showed a strong control effect on *Solanum nigrum*. *Alternaria* methyl ether (AME) from *Alternaria* MGTMMP31 exhibited significant anti-proliferative activity against human HCC cells (HUH-7) in vitro and in vivo [16].

As the source of the Three Rivers, protection of the ecological environment in the Tibetan Plateau is of vital importance. This region has special ecological characteristics such as a high altitude, strong radiation, aridity, and large temperature differences between day and night [17], which lead to a very low environmental carrying capacity [18], and its ecological environment is extremely fragile and prone to damage. In Qinghai Province, 67 species of farmland weeds belonging to 25 families have been documented, and the extensive use of chemical herbicides has led to ecological pollution and weed resistance in the Qinghai-Tibetan Plateau region [19]. In view of its special ecological conditions, reducing the amounts of chemical herbicides used and developing biological herbicides can effectively reduce the pollution of the ecological environment, which is compatible with the demand for the sustainable development of modern agriculture. This study describes a new species of *Alternaria* isolated from naturally diseased *Medicago sativa* L., and its development and selection as a promising microorganism for weed control were studied. The aim is to provide a theoretical basis for further research and the development of new and efficient herbicides in the future, thereby effectively filling the existing gap in the field of microbial herbicide research in the Tibetan Plateau.

## 2. Materials and Methods

### 2.1. Test Strain

Strain GD-011 was isolated from naturally diseased *M. sativa* roots by tissue isolation methods [20]. It is preserved in the China General Microbiological Culture Collection Center, with a preservation address of No. 3, No. 1 Yard of Beichen West Road, Chaoyang District, Beijing, and a preservation number of CGMCC No. 40558.

### 2.2. Test Weeds and Crops

The tested species included common weeds in the fields such as *C. album* (*Chenopodium album* L.), *E. densa* (*Elsholtzia densa* Benth), *M. verticillata* (*Malva verticillata* L.), *P. lapathifolium* (*Polygonum lapathifolium* L.), *A. retroflexus* (*Amaranthus retroflexus* L.), *A. fatua* (*Avena fatua* L.), *T. arvense* (*Thlaspi arvense* L.), *P. aviculare* (*Polygonum aviculare* L.), and *G. aparine* (*Galium aparine* L.); the main crops in the Tibetan Plateau of *H. vulgare* (*Hordeum vulgare* L.), *T. aestivum* (*Triticum aestivum* L.), *B. napus* (*Brassica napus* L.), *V. faba* (*Vicia faba* L. )and *P. sativum* (*Pisum sativum* L.).

### 2.3. Test Design and Method

#### 2.3.1. Pathogenicity of Biocontrol Strain GD-011 to Weed Leaves

Leaves of the weeds *C. album*, *E. densa*, *M. verticillata*, *P. lapathifolium*, *A. retroflexus*, *A. fatua*, *T. arvense*, *P. aviculare*, and *G. aparine* were collected from the experimental field and brought into the lab. Then, their surface was washed, and the weeds were disinfected with 75% alcohol for 30 s, washed three times with sterile water, and naturally dried. Samples were then placed on a Petri dish (Φ = 9 cm) padded with sterile filter paper, with one piece of filter paper per dish; sterile water was used to wet the filter paper to provide a moist environment. Pieces of hyphae (Φ = 8 mm) were taken from the edges of colonies of strain GD-011 cultured on PDA medium for 7 days at 25 °C and used to inoculate the adaxial surfaces of the leaves. Inoculated sterile PDA medium samples were used as controls, and each treatment was repeated three times. The dishes were cultured in an incubator with light for 12 h and dark for 12 h (12L:12D) at (25 ± 1) °C. After 7 days, the lesion area was measured and calculated as 1/4 × length × width × 3.14.

#### 2.3.2. Pathogenicity of Biocontrol Strain GD-011 to Potted Weeds and Crop Safety Pathogenicity to Potted Weeds

The weeds *C. album*, *E. densa*, *M. verticillata*, *P. lapathifolium*, *A. retroflexus*, *A. fatua*, *T. arvense*, *P. aviculare*, and *G. aparine* in the 4–5 leaf stages and normal growth conditions were transplanted into pots (Φ = 15 cm) and cultured at room temperature (25 ± 1 °C) for 1 week. Pieces of hyphae were inoculated in PDB culture medium (250 mL/bottle), at five pieces per bottle, and shaken and cultured for 120 h at 25 °C and 180 r/min. Then, the fermentation broth was filtered through four layers of sterile gauze to obtain the fermentation filtrate with a spore concentration of 1.0 × 10^8^ mycelial fragments/mL. It was inoculated on the normally growing potted weed plants at the 4–7 leaf stage for 3 days by spray inoculation, and the inoculation amount was 25 mL/pot. After the inoculated weed plants were cultured in plastic bags for 24 h, they were placed in an artificial climate box at 25–30 °C and L:D = 12 h:12 h, and each treatment was repeated three times. Plants inoculated with sterile PDB medium were used as controls. After 7 days, the disease incidence of inoculated weeds was determined, and the incidence rate and fresh weight control effect were calculated by the following equations [21]:

$$\mathrm{Fresh}\;\mathrm{weight}\;\mathrm{effect}\;(\%)=\frac{\mathrm{Control}\;\mathrm{fresh}\;\mathrm{weight}-\mathrm{Processed}\;\mathrm{fresh}\;\mathrm{weight}}{\mathrm{Control}\;\mathrm{fresh}\;\mathrm{weight}}\times 100\%$$

$$\mathrm{Incidence}\;\mathrm{rate}=\frac{\mathrm{Number}\;\mathrm{of}\;\mathrm{diseased}\;\mathrm{leaves}}{\mathrm{Number}\;\mathrm{of}\;\mathrm{leaves}\;\mathrm{investigated}}\times 100\%$$

$$\mathrm{Disease}\;\mathrm{index}=\frac{\mathrm{Number}\;\mathrm{of}\;\mathrm{diseased}\;\mathrm{leaves}\times \mathrm{Number}\;\mathrm{of}\;\mathrm{corresponding}\;\mathrm{levels}}{\mathrm{Total}\;\mathrm{number}\;\mathrm{of}\;\mathrm{leaves}\;\mathrm{investigated}\times \mathrm{Number}\;\mathrm{of}\;\mathrm{highest}\;\mathrm{levels}}\times 100\%$$

##### Crop Safety

Five main crops in Qinghai, *V. faba*, *P. sativum*, *H. vulgare*, *T. aestivum*, and *B. napus*, were planted in pots with Φ = 12 cm and cultured indoors. After dilution, the fermentation broth of the strain was inoculated on the crop plants at the 3–6 leaf stages using the weed pathogenicity method described above. Each treatment was repeated three times, and crops inoculated with sterile PDB medium were used as controls. After 7 days, the disease incidence was determined. The safety evaluation criteria of crops were as follows: NS means that the plants have no susceptibility (no disease spots, normal growth); LS indicates light susceptibility (scattered patches on the leaves and slightly reduced growth and development); MS indicates moderate susceptibility (one-fifth to one-fourth of the leaf area has disease spots, and the growth is inhibited); SS indicates severe susceptibility (a large number of plants die and their growth and development are seriously inhibited).

#### 2.3.3. Identification of Biocontrol Strain GD-011

##### Morphological Identification

The strain was placed on a PDA plate and cultured in an incubator at 25 °C with alternating L:D = 12 h:12 h. The growth rate, colony shape, and color changes were observed, and the morphology of hyphae and spores was observed under an optical microscope. Preliminary identification was carried out in combination with the Fungus Identification Manual [22].

##### Molecular Identification and Phylogenetic Tree Construction

The genomic DNA of the strain was extracted by the CATB method [23]. PCR amplification was performed with universal primers ITS1 and ITS4 [24], EF1-728F and EF1-986R [25], and Alt-for and Alt-rev [26], all of which were synthesized by Shanghai Sangon Biotech Co., Ltd. (Shanghai, China). The PCR reaction systems all consisted of 25 µL, comprising 0.5 µL of forward and reverse primers (10 mol/L), 0.5 µL of DNA template, 2.5 µL of 10 × PCR buffer, 2.5 µL of Taq enzyme, and 18.5 µL of dd H_2_O. The PCR cycle settings were as follows: pre-denaturing at 94 °C for 5 min, denaturing at 94 °C for 45 s, annealing at 55 °C for 45 s, and extending at 72 °C for 1 min, for 30 cycles, then a final extension at 72 °C for 10 min, and heat preservation at 4 °C. After the amplification was completed, electrophoretic detection was conducted, and the purified product was recovered using the SanPrep column DNA J gel recovery kit (SK8131, Shanghai Sangon, Shanghai, China) and sent to Shanghai Sangon for two-way sequencing. Phylogenetic relationships were based on the analysis of ITS-EF-1a-Alt a1 matrix gene sequences. All sequences downloaded from NCBI’s GenBank sequence database in this study were concatenated by FASTA alignment and imported into BioEdit to be compared and analyzed. Preliminary alignments of the multiple sequences were conducted using CLUSTAL X 1.8, with manual adjustment using BioEdit for visual improvement where necessary [27,28]. A phylogenetic tree was constructed by neighbor-joining using MEGAX 7.0 with 1000 bootstrap replications.

##### Scanning Electron Microscopic Observations of the Infection Process of the Strain on *E. densa* Leaves

The representative weed in the farmland of Qinghai, *E. densa,* was selected as the test subject. Filter paper was laid in a sterilized Petri dish (Φ = 90 mm), and *E. densa* leaves were placed on it. The filter paper was soaked with sterile water to provide a moist environment, a sample of strain cake (Φ = 8 mm) was placed in the center of the leaves with a punch, and a sterile PDA strain cake was used as a control. Each treatment and the control were repeated three times. The experiment was carried out at 25–28 °C. After inoculation, samples were taken every day for 7 days to observe the invasion process of the hyphae.

After pieces of the strain hyphae were inoculated for 1–7 days, 3–6 leaf segments (0.5–1.0 cm) were prepared with fresh razor blades from each inoculated leaf. They were fixed in 2.5% (*v*/*v*) glutaraldehyde in 0.1 M phosphate buffer (pH 7.2) under vacuum for 2 h at room temperature and then in the same fixation buffer at 4 °C. The leaf samples were washed three times with 0.1 M phosphate buffer (pH 7.2) and dehydrated by a fractional ethanol series (70%, 80%, 90%, and 100%) for 30 min in each gradient concentration. The samples were dried with liquid CO_2_ at the critical point. The fixed material was coated with a 10 nm layer of gold/palladium and observed by scanning electron microscopy (Nikon ECLIPSE E100, Shanghai Danding International Trading Corporation, Shanghai, China).

#### 2.3.4. Statistical Analysis

Excel and SPSS 25.0 were used to statistically analyze the experimental data. For single-factor statistical analysis, Duncan’s new complex difference method was used for analysis of variance, and the *p* value was used to describe the significant differences in the data.

## 3. Results

### 3.1. Pathogenicity of Alternaria Gaisen GD-011 to Weed Leaves

**Figure 1:**
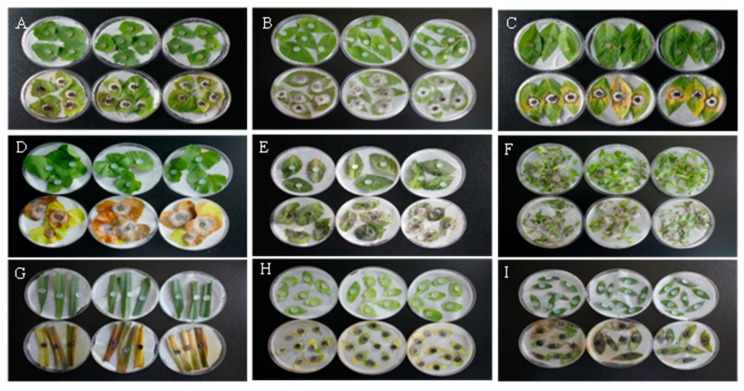
Pathogenicity of strain GD-011 to weed leaves in vitro. (**A**) *C. album*; (**B**) *E. densa*; (**C**) *P. lapathifolium*; (**D**) *M. verticillata*; (**E**) *A. retroflexus*; (**F**) *G. aparine*; (**G**) *A. fatua*; (**H**) *T. arvense*; (**I**) *P. aviculare.* Note: Leaves in the top row of A to I were the controls, while those in the bottom row of A to I were treatments to test the pathogenicity of GD-011 to different weeds in vitro.

As shown in Figure 1, 7 days after inoculation with the GD-011 strain cake in vitro, the leaves were damaged. The leaves of *C. album*, *P. lapathifolium*, and *M. verticillata* were yellow and green, and hyphae had penetrated and grown from adaxial to abaxial surface on the leaves at the inoculation site. Leaves of *E. densa*, *G. aparine*, and *A. retroflexus* had gray hyphae and showed withering symptoms in the later stage. Leaves of *A. fatua*, *T. arvense*, and *P. aviculare* had faded and turned yellow and then black 7 days after inoculation. Infestation of *M. verticillata* produced the largest spot area of 5.78 cm^2^, and infestation of *G. aparine* produced the smallest spot area of 0.52 cm^2^ (Table 1). The pathogenicity sequence of GD-011 hyphae to different weeds in vitro is as follows: *M. verticillata* > *E. densa* > *P. aviculare* > *G. aparine* > *P. lapathifolium* > *C. album* > *A. retroflexus* > *A. fatua* > *T. arvense*.

### 3.2. Pathogenicity of A. gaisen GD-011 to Potted Weeds

**Figure 2:**
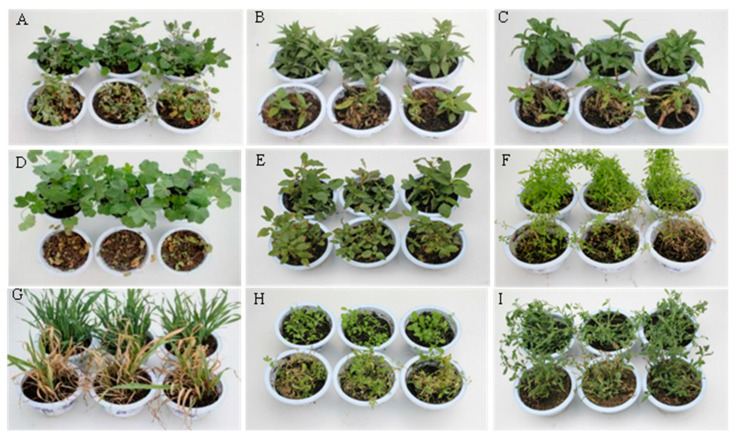
Pathogenicity of strain GD-011 to potted weeds. (**A**) *C. album*; (**B**) *E. densa*; (**C**) *P. lapathifolium*; (**D**) *M. verticillata*; (**E**) *A. retroflexus*; (**F**) *G. aparine*; (**G**) *A. fatua*; (**H**) *T. arvense*; (**I**) *P. aviculare*. Note: Leaves in the top row of A to I were the controls, while those in the bottom row of A to I were treatments to test the pathogenicity of GD-011 to different potted weeds.

After the GD-011 fermentation products were sprayed for 7 days, the incidence rate and fresh weight control effect on *M. verticillata* reached 94.83 and 90.81%, the incidence rates of *C. album*, *E. densa*, and *P. lapathifolium* were 75.5, 80.87, and 77.13%, respectively, with leaves that were curly, and the lower leaves were wilting and yellow. After 7 days, the leaves of *M. verticillata* lost their green coloration and showed necrosis and shedding; while the leaves of *A. fatua* turned yellow, and two-thirds of them had died, with an incidence rate of 82.13%. The leaves of *A. retroflexus* and *P. aviculare* were sporadically spotted, the stems and leaves were black and withered, and the incidence rates were 56.2 and 42.07%, respectively. The disease index values showed that *M. verticillata*, *G. aparine*, *E. densa*, and *A. fatua* were the most sensitive to the fermentation filtrate of the strain. After 7 days, the inoculated weeds did not recover, and the disease symptoms worsened until the whole pot of weeds died (Figure 2 and Table 2).

### 3.3. Safety of A. gaisen GD-011 for Crops

**Figure 3:**
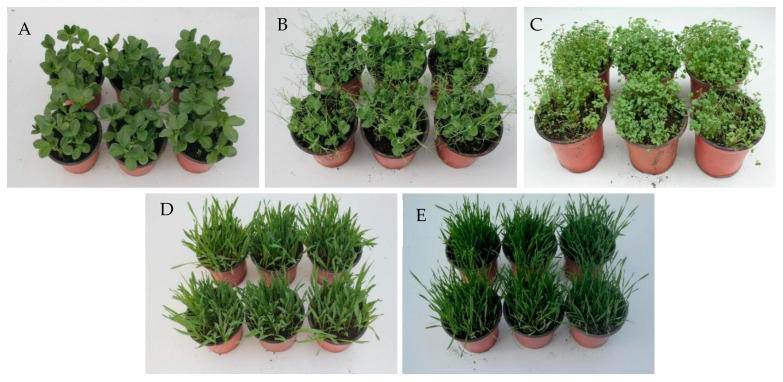
Safety of strain GD-011 to crops. (**A**) *V. faba*; (**B**) *P. sativum*; (**C**) *B. napus*; (**D**) *H. vulgare*; (**E**) *T. aestivum.* Note: Leaves in the top row of A to E were the controls, while those in the bottom row of A to E were treatments to test the safety of strain GD-011 to crops.

The fermentation broth of strain GD-011 was not pathogenic to *P. sativum*, *V. faba*, or *T. aestivum*. The growth and plant heights of these crops were not affected compared with the control plants, and they grew healthy, showing no response (NS). *Brassica napus* showed susceptibility, where 25% of the leaves had black spots on the leaf edges, and plants showed a wilting phenomenon, indicating that they were moderately susceptible (MS). The filtrate was slightly pathogenic to *H. vulgare*, in which a small number of leaves had sporadic spots on the veins, and the leaves were yellow, showing a slight reaction (LS) overall (Figure 3).

### 3.4. Morphological Identification of A. gaisen GD-011

**Figure 4:**
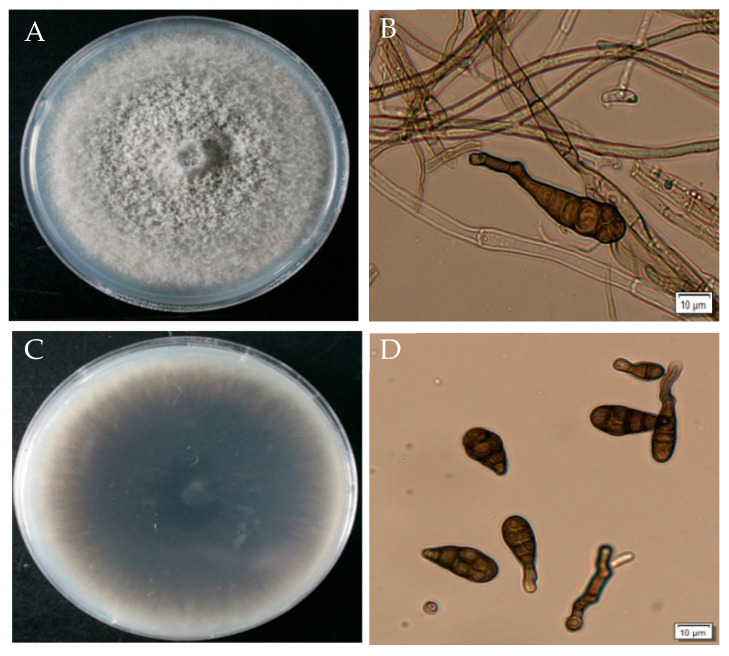
Morphological characteristics of strain GD-011. (**A**,**C**) Morphology of strain GD-011 on a PDA plate; (**B**) conidiophore; (**D**) conidia.

GD-011 colonies are white at the beginning on the PDA plate and gradually develop into olive or dark green velvet in the later stage, with neat edges. The aerial hyphae are dense. The hyphae are colorless and septate. Conidia appear dark brown under the light microscope and have an inverted rod shape with a transverse diaphragm and longitudinal septum on the surface. The transverse septum is thicker, and the number of transverse septa is 3–5. The septa slightly overflow and contract, and the beak is cylindrical or conical. According to the cultural and morphological characteristics of the strain, the pathogen was initially identified as *Alternaria* sp. (Figure 4).

### 3.5. Molecular Biological Identification of A. gaisen GD-011

**Figure 5:**
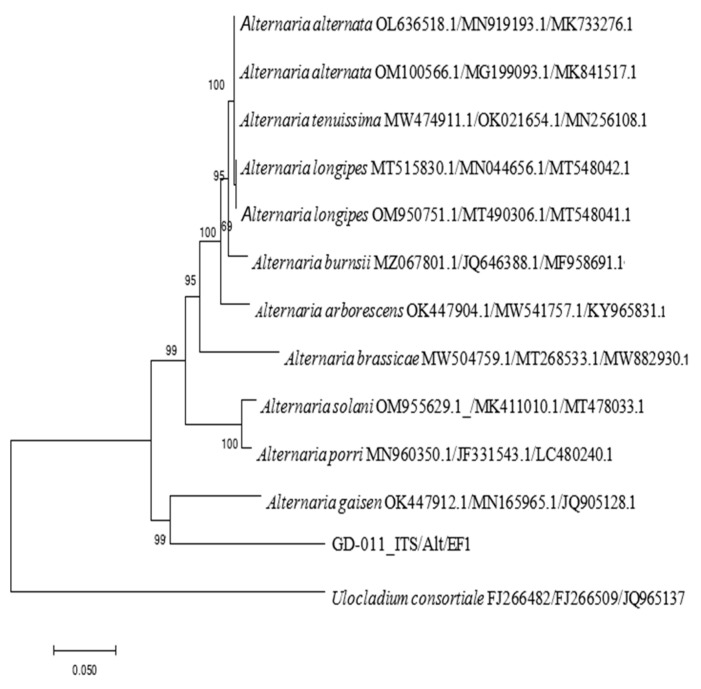
Phylogenetic tree of GD-011 based on the *rDNA-ITS*, *EF-1a*, and *Alt a1* gene sequences.

The rDNA-ITS, *EF-1α*, and *Alt a 1* gene sequences of strain GD-011 were amplified by PCR, and three gene fragments with lengths of 533 bp, 264 bp, and 477 bp were obtained, respectively. According to the sequences of the strain, a phylogenetic tree of the strain was constructed using *Ulocladium consortiale* as an outgroup (Figure 5). GD-011 and *A. gaisen* are clustered together on the phylogenetic tree, and the support rate is 99%. Note that *A. gaisen* may be accurately separated from the other species through these three gene sequences, showing good conservation overall. According to the phylogenetic analysis of the three gene sequences and morphological characteristics of GD-011, strain GD-011 was identified as *Alternaria gaisen*.

### 3.6. Observations on the Pathogenic Process in E. densa by Strain GD-011

**Figure 6:**
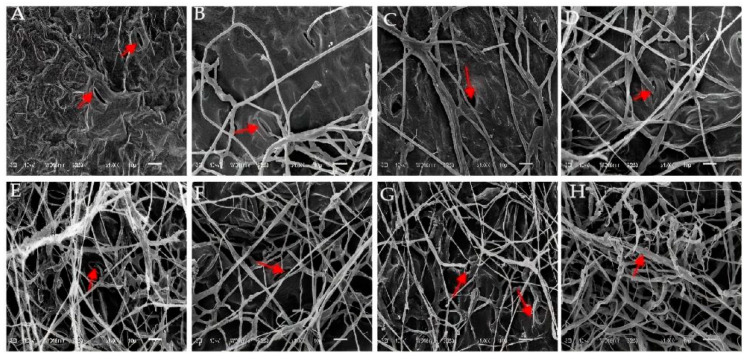
Characteristics of the invasion of strain GD-011 into the leaf tissue of *E. densa* by scanning electron microscopy. (**A**) Not inoculated; (**B**,**C**) inoculation for 1–2 days; (**D**,**E**) inoculation for 3–4 days; (**F**,**G**) inoculation for 5–6 days; (**H**) inoculation for 7 days; s, stomata; H, hyphae; ih, invasive hyphae; td, tissue destruction.

Figure 6 shows the ultrastructure of *E. densa* leaves as affected by strain GD-011. The cell structure of uninoculated healthy *E. densa* leaves is normal, and the tissues are arranged neatly (Figure 6A). After 1–2 days of inoculation, hyphae penetrate the stomata, and small hyphae are formed around them (Figure 6B,C). After 3–4 days, many hyphae are attached to the tissue surface, and the leaf tissue is damaged (Figure 6D,E). After 5–6 days, as the hyphae form a fungal net and parasitize the tissue surface, the parasitic tissue of the hyphae absorbs nutrients, and the plant tissue becomes diseased (Figure 6F,G). With the infection of many hyphae, the tissue surface shows obvious destruction. After 7 days, the hyphae grow vigorously all over the tissue surface, the metabolism of the *E. densa* leaf tissue becomes disordered, and the infected cells gradually die (Figure 6H).

## 4. Discussion

Studies have shown that *Alternaria* toxin can increase the permeability of plant cell membranes, enhance the peroxidation of cell membrane lipids, and then cause plant death [14]. Therefore, *Alternaria* and its metabolites have the potential to be developed as microbial herbicides. The strain GD-011 isolated from the roots of *M. sativa* in Guide County, Qinghai Province, showed varying degrees of control effects on nine species of weeds by preliminary screening in vitro and the re-screening of live potted plants. The pathogenicity of strain GD-011 to the leaves of the nine weeds varied from strong to weak as follows: *M. verticillata > E. densa > P. aviculare > G. aparine > P. lapathifolium > C. album > A. retroflexus > A. fatua > T. arvense*. The in vivo spray inoculation test showed that strain GD-011 has a strong pathogenic effect on the weeds *M. verticillata*, *E. densa*, and *A. fatua*, with incidence rates of more than 80.87% and fresh weight control effects of more than 71.8%. Crop safety tests showed that strain GD-011 causes moderate symptoms in *B. napus* seed crops and has slight impacts on *H. vulgare*, but it is relatively safe for *T. aestivum*, *P. sativums*, and *V. faba*. According to its morphology combined with molecular identification using *rDNA-ITS* and *EF-1α* jointly constructed with the *Alt a1* gene, GD-011 was identified as *A. gaisen*. From the perspective of herbicide development, its metabolites have good inhibitory effects on most weeds and are relatively safe for three local crops. Therefore, *A. gaisen* GD-011 can be safely used in *T. aestivum*, *P. sativum*, and *V. faba* crop fields that have *M. verticillata*, *E. densa*, and *A. fatua* as the dominant weeds.

When herbicides act on weeds, the weeds first respond to the herbicide themselves in the early stages, followed by symptoms such as wilting, slowing growth, curling leaves, water loss, and even death. Scanning electron microscopic observations showed that the mycelium of strain GD-011 invaded the leaf tissue through the stomata on the surface. Then, numerous mycelia became attached to the surface of the tissue, with the formation of a parasitic mycelium network on the surface of the tissue. The metabolism of *E. densa* leaf tissues then became disturbed, and leaf tissues appeared to be broken.

This study reports the first evaluation of the herbicidal activities of *A. gaisen* against weed species, so there is an open field of research for its use as a potential bioherbicide candidate. In the future, further research can be conducted on the safety of GD-011 for other crops and vegetables to clarify its safety and applicability and provide a theoretical basis for the further development and utilization of microbial agents. In addition, more in-depth and systematic research is needed on the herbicide mechanism, field validation, and formulation development of strain GD-011, in order to provide alternative resources for the research and development of natural herbicides.

## 5. Conclusions

*Alternaria gaisen* GD-011 isolated from naturally susceptible *M. sativa* roots was found to be effective in controlling nine species of weeds in Qinghai farmland, and the fermentation filtrate was found to be very safe for *P. sativum*, *V. faba*, and *T. aestivum*. GD-011 caused moderate symptoms in *B. napus* seed crops and had a slight impact on *H. vulgare*. From the perspective of herbicide development, its metabolites have good inhibitory effects on most weeds and are relatively safe for crops. The strain was identified as *A. gaisen* based on morphology and a combined analysis of multiple gene sequences. The results showed that *A. gaisen* GD-011 can be safely used in *T. aestivum*, *P. sativum*, and *V. faba* crop fields that have *M. verticillata*, *E. densa*, and *A. fatua* as the dominant weeds. Therefore, this strain can effectively and safely control most broad-leaved weeds with a low cost, no pollution, and low residues.

This study investigated the pathogenicity, crop safety, morphological and molecular identification, and infection process of strain GD-011 and is the first report using the fungus *A. gaisen* for weed control.

## Figures and Tables

**Table 1:** Symptoms of leaves of nine weeds after inoculation with GD-011.

Weed Species	Incidence Area/cm^2^	Incidence Characteristics
*Chenopodium album*	0.49 ± 0.25	Leaf inoculation site produces a few gray mycelia, leaf yellowing, mycelia have penetrated the leaves
*Elsholtzia densa*	2.45 ± 0.16	Mycelium covering the entire leaf blade, leaf blade with chlorosis
*Polygonum lapathifolium*	3.27 ± 0.33	Leaves turn green and yellow, spots make the whole leaf wither and turn yellow-brown, leaf inoculation site covered with mycelium
*Malva verticillata*	5.78 ± 0.25	Spots appear first, then yellowing, and eventually the leaf color becomes yellowish brown, with numerous mycelia on the surface
*Amaranthus retroflexus*	3.56 ± 0.71	Produces dark brown irregular spots, mycelium makes the whole leaf yellow and wrinkled
*Gallium aparine*	0.52 ± 0.08	Gray mycelium production at the inoculation site, followed by leaf chlorosis
*Avena fatua*	3.41 ± 0.18	Leaves turn green and yellow

**Table 2:** Pathogenicity of the fermentation filtrate of strain GD-011 to different weeds in vivo.

Weed Species	Incidence Rate/%	Disease Index	Fresh Weight Control Effect/%
*C. album*	75.5 ± 2.37 b	61.4 ± 3.14 c	60.2 ± 2.65 bc
*E. densa*	80.87 ± 2.38 ab	81.87 ± 2.96 b	83.9 ± 1.63 a
*P. lapathifolium*	77.13 ± 4.70 ab	59.23 ± 1.81 cd	52.47 ± 2.36 cd
*M. verticillata*	94.83 ± 1.66 a	94.47 ± 2.38 a	90.81 ± 1.41 a
*A. retroflexus*	56.2 ± 6.18 cd	43.4 ± 2.50 e	45.27 ± 2.43 d
*G. aparine*	68.5 ± 13.05 bc	76.1 ± 5.78 b	66.5 ± 3.67 b
*A. fatua*	82.13 ± 1.38 ab	84.03 ± 3.29 ab	71.8 ± 7.52 b
*T. arvense*	41.23 ± 0.54 d	48.73 ± 3.19 de	44.43 ± 3.38 d
*P. aviculare*	42.07 ± 3.24 d	42.17 ± 2.22 e	46.9 ± 1.15 d

Note: Different lowercase letters indicate significant differences based on the least significant difference test (*p* < 0.05).

## Data Availability

Data are contained within the article.
